# Overflow in science and its implications for trust

**DOI:** 10.7554/eLife.10825

**Published:** 2015-09-14

**Authors:** Sabina Siebert, Laura M. Machesky, Robert H. Insall

**Affiliations:** Adam Smith Business School, University of Glasgow, Glasgow, United Kingdom; CRUK Beatson Institute for Cancer Research, University of Glasgow, Glasgow, United Kingdom and College of Medical, Veterinary and Life Sciences, University of Glasgow, Glasgow, United Kingdom; CRUK Beatson Institute for Cancer Research, University of Glasgow, Glasgow, United Kingdom and College of Medical, Veterinary and Life Sciences, University of Glasgow, Glasgow, United KingdomR.Insall@beatson.gla.ac.uk

**Keywords:** point of view, reproducibility, overflow, trust in science, scientific conduct

## Abstract

To explore increasing concerns about scientific misconduct and data irreproducibility in some areas of science, we interviewed a number of senior biomedical researchers. These interviews revealed a perceived decline in trust in the scientific enterprise, in large part because the quantity of new data exceeds the field's ability to process it appropriately. This phenomenon—which is termed ‘overflow’ in social science—has important implications for the integrity of modern biomedical science.

In recent years scientists, academic journal editors and the press have all expressed concerns about the soundness of scientific research. These concerns have led to questions about the reliability of science among the general public and within the scientific community itself. Particular themes include the low reproducibility of published findings in certain fields (Begley and Ellis, 2012; Nature Biotechnology, 2012; Button et al., 2013; Fishburn, 2014; Nuffield Council on Bioethics, 2014), ineffective peer review processes (Eisen, 2011), increasing rates of retractions of papers from academic journals (Steen, 2011), and/or reluctance to publish negative results (Prinz et al., 2011). Some commentators attribute these problems to a lack of scientific rigour or, in some cases, fraud (Resnik and Dinse, 2013).

Multiple projects are currently objectively assessing whether the reproducibility of results is declining (Open Science Foundation, 2012; Errington et al., 2014). Here we instead explore the subjective question of how the perceived decline in reproducibility and integrity has affected trust in the scientific enterprise. Biomedical science depends on a large network of trust among individuals and organisations, including the accurate reporting of data and observations, and the rigorous peer reviewing of publications and grant applications. Thus changes in the way individual scientists trust each other, or the enterprise as a whole, have major implications for the future of research.

Changes in the way individual scientists trust each other, or the enterprise as a whole, have major implications for the future of research.

## Research design

We designed a qualitative study, which involved collecting data by semi-structured interviews with 20 prominent principal investigators in the US, each with between 20 and 60 years of experience of basic biomedical research. Seven of the interviewees were government employees, and thirteen worked within universities. Our questions were grouped into four sections: (1) Have the scientists interviewed observed a decrease in the trustworthiness of science in their professional community and, if so, what are the main factors contributing to these perceptions? (2) How do the increasing concerns about the lack of robustness of scientific research affect trust in research? (3) What concerns do scientists have about science as a system? (4) What steps can be taken to ensure the trustworthiness of scientific research?

Interview transcripts were analysed to identify common themes relating to trust between individuals, within organizations, and in bodies of knowledge such as journals and the published literature. A detailed analysis of the interviews will be submitted to an appropriate social science journal when it is complete.

## The concept of overflow

Our data reflected widespread concerns about scientific integrity, such as the low reproducibility rate in some fields, professional pressures to publish in top-rated journals, and the bad publicity surrounding the retraction of papers from academic journals. One issue that was downplayed by the majority of our interviewees was the incidence of fraud. Fraud, in their view is very serious, but it happens extremely rarely. Interviewees were more concerned about researchers overstating their findings or misrepresenting their data. What our study revealed, and what is not discussed in the scientific literature, is that concerns within the scientific community often related to what social scientists refer to as ‘overflow’.

Overflow (also referred to as surplus, excess or overspill) is seen as the explicit opposite of scarcity. It is a concept used in economic theory, management, consumer studies and politics, though these disciplines have different interpretations of what really constitutes overflow. For example, the notion of ‘having too much of something’ underpins the study of the economics of attention (Lanham, 2006), which addresses how people choose which subjects to prioritize when there is too little time and too much information. Overflow can be construed as both positive (more means better) and negative, but when it is observed most authors agree that overflow must be *managed* (Czarniawska and Löfgren, 2012). The concept also evokes the image of a mess that needs to be dealt with, or waste that needs to be removed.

Social scientists have looked into the ways in which overflow is managed in public and private organizations, by professions, and by individuals (Czarniawska and Löfgren, 2012, 2013). For example, in news agencies overflow is managed through selection, which is inherent in the profession of journalism (Czarniawska, 2012). Gatekeepers who are experts in a given field rapidly and exactly apply their judgment (guided by appropriate criteria) to select the information that should be published. When overflow increases, the number of gatekeepers must also increase.

In healthcare the prevalent method of managing the ever-increasing number of patients is prioritization, and the development of “overflow devices” such as the introduction of waiting time guarantees or measures to enable patient mobility (Norén, 2013). Generating overflow devices often involves changing the ways in which markets are regulated. For example, enabling patient mobility allows patients to be moved from inefficient to efficient providers.

Another example of overflow is information overload, which is often described as being “swamped with information, but starved of data”. Ways in which information overload can be tackled include indexing, categorizing, codifying and archiving information (Fellman and Popp, 2013; Löfgren, 2013).

## What does overflow in science mean?

Interview accounts were redolent with overflow, with scientists frequently raising concerns over the rapid proliferation of journals, and the immense number of papers being published in relatively new mega-journals such as PLOS ONE. Our interviewees also commented on a striking increase in the number of grant applications submitted to grant awarding bodies and in the number of applications for jobs. One scientist talked about “increased pressure in the funding arena”, while another stated that “the resources available per scientist are a lot less which makes everything much more competitive”. This increase in competition for resources is seen by one interviewee as detrimental to science:

Publications and research have grown exponentially so if you go back twenty-five/thirty years ago when I was in training there were less journals, the literature was smaller, now there is increased number of journals, more people competing for smaller resources so it seems like there's a lot that gets published that is pretty devious or questionable.

One interviewee complained about overflow in relation to the newly created journals:

There's this proliferation of journals, a huge number of journals… and I tend not even to pay much attention to the work in some of these journals. (…) And you're always asked to be an editor of some new journal. (…) I don't pay much attention to them.

The exponential growth of scientific outputs stands in contrast with the artificial scarcity (Young et al., 2008) of prestigious publication outlets (Eisen, 2015). Also, the increase in job and grant applications coincides with a perceived decrease in the funding available for science which, in turn, leads to increased competition among scientists for a diminishing pool of jobs and funds (Bourne, 2013; Alberts et al., 2015). When there is an overflow of applications for postdoctoral or faculty jobs, high impact publications often become the proxy for having discovered something significant in one's field, despite the strong evidence that it is a flawed measure (Seglen, 1997; Curry, 2012). This propagates a vicious cycle in which the search for publication impact is seen as the only goal of science. One scientist observed:

This propagates a vicious cycle in which the search for publication impact is seen as the only goal of science.

What some scientists are frustrated by is that we've let the publishing companies and the impact factor take over our science (…) it's a motivating force for post-docs. Some of them come in [and say] ‘if I don't get a Cell, Science or Nature I'm not going to get a faculty position’. And that comes from the impact factor hype.

## Is overflow a problem?

Overflow in science appeared to be associated with quality control problems. Interviewees suggested that the rapid proliferation of scientific outputs was inconsistent with the capacity of the world of science to verify the quality of outputs. The number of scientists/authors is dramatically increasing, whereas the number of reviewers qualified to assess the scientific outputs does not increase proportionally. That good reviewers are a scarce resource was a sentiment often expressed by our interviewees who had experience as journal editors. And those reviewers, who are often based in the most established labs and institutes, are already over-committed and reluctant to review papers from new, less well-known journals.

The size of an average scientific article has also increased in recent years. One interviewee compared papers published in the 1970s with those published nowadays and concluded that today's papers are based on much richer data, and contain many more figures and tables.

**Figure 1:**
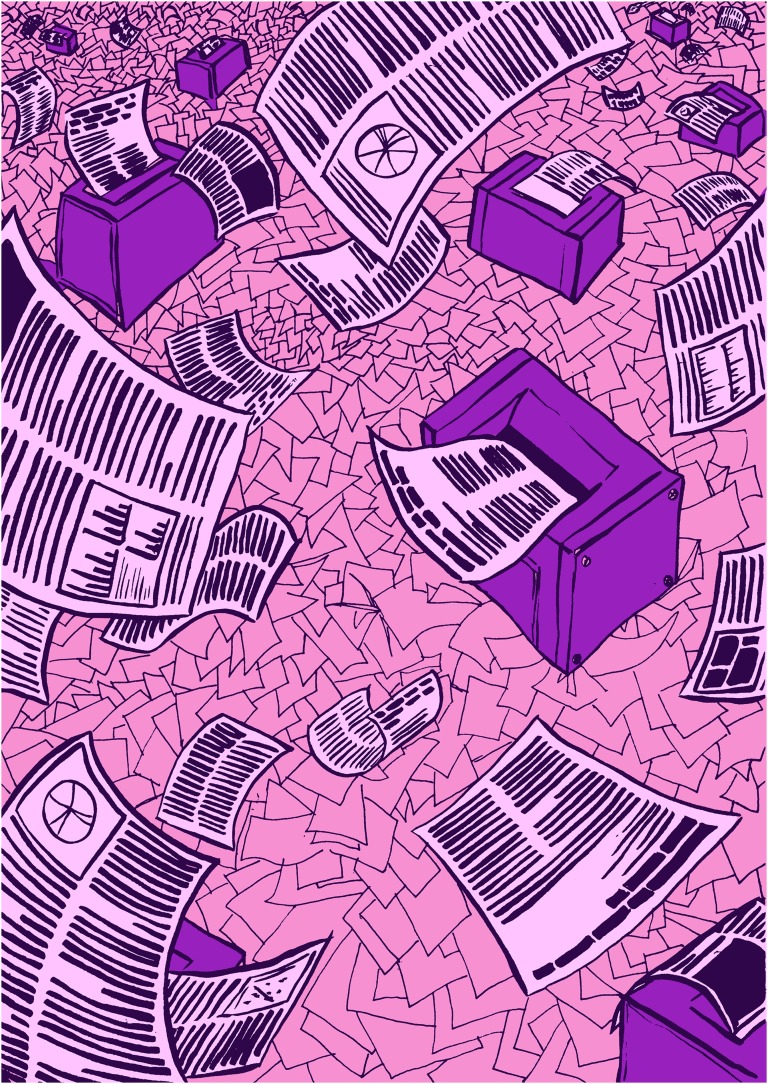
Concerns within the scientific community about scientific integrity and peer review are often related to what social scientists refer to as “overflow”.

I tell people in my lab go and (…) pick up a copy of Cell from the 70s or Journal of Cell Biology. (…) Papers that were published then, would be Figure 1A of a Cell paper today. (…) There's so much more data and information that has to go into each paper, not just for a Cell paper but for a paper in any journal. So easily five times and probably ten or twenty times more data than there was in the past.

Another interviewee commented on the “Figure 7 phenomenon”, where scientists feel compelled to expand their papers by adding striking information that is likely to make their paper stand out:

In my lab we laugh about ‘Figure 7’ in a Cell paper and it's the figure that (…) got them into that journal that had something sexy that gives it a twist. Up until then maybe the paper is pretty solid and reliable and then they have a Figure 7 (…) that brings in something that's trendy.

Another interviewee echoed this observation:

For me the bigger concern is (…) trying to make it a hot, sexy story to try to get into a high tier journal. For me that's a bigger problem because it creates pressure on junior people (…) to feel that they will only get a job if they publish in Nature or Science or Cell and if they don't publish there they're washed-up, useless, a failure.

One interviewee further commented on the dangers of over-inflating the findings:

The temptation to inflate their findings or exaggerate their findings might be a little bit greater but then of course the bigger you are the harder you fall, so if you over-inflate or conflate your results too much and then all of a sudden someone catches you on it then it's a bigger distance to fall.

In general, interviewees agreed that there was an issue with the soundness of the scientific literature, but they clearly believed that the problem was not overt fraud (which they felt is rare and overstated) but a relaxation of rigour, driven by pressure to be visible in a competitive climate.

Interviewees agreed that there was an issue with the soundness of the scientific literature, but they clearly believed that the problem was not overt fraud (which they felt is rare and overstated) but a relaxation of rigour, driven by pressure to be visible in a competitive climate.

## The role of reputation

Echoing the problems captured in the literature on the economics of attention (Lanham, 2006), our interviewees complained about not being able to read everything that was published even in their own narrow disciplines. When faced with the “flood” of scientific studies, they were forced to be selective, so familiarity with the personal reputation of the authors or the journal often affected their decisions about whether to trust them. One interviewee commented on the role of personal reputation:

There are some people that I know to be really rigorous scientists whose work is consistently well done (…). If a paper came from a certain lab then I'm more likely to believe it than another paper that might have come from a different lab whose (…) head might be somebody that I know tends to cut corners, over-blows their conclusions, doesn't do rigorous experiments, doesn't appreciate the value of proper controls.

This appears to suggest that personal reputation becomes a proxy for trustworthiness, with some scientists trusting science produced in reputable labs by reputable scientists.

In addition to influencing which papers scientists decide to read and how much trust they place in what they read, reputation also influences the peer review process. Although all interviewees described being equally rigorous in the assessment of all papers in the peer review process, some suggested that it is natural to trust someone who has a good reputation. One interviewee suggested:

If I know that there's a very well established laboratory with a great body of substantiated work behind it I think there is a human part of me that is inclined to expect that past quality will always be predicting future quality I think it's a normal human thing. I try not to let that knee–jerk reaction be too strong though.

One interviewee suggested that not knowing the scientists behind the study might make them “look more carefully” at the data:

If I don't know the authors then I will have to look more carefully at the data and (…) evaluate whether (…) I feel that the experiments were done the way I would have done them and whether there were some, if there are glaring omissions that then cast out the results (…) I mean [if] I don't know anything I've never met the person or I don't know their background, I don't know where they trained (…) I've never had a discussion with them about science so I've never had an opportunity to gauge their level of rigour…

Another interviewee expressed scepticism about the rapid proliferation of new journals:

The journal that [a paper] is published in does make a difference to me, … I'm talking about (…) an open access journal that was started one year ago… along with five hundred other journals, (…) literally five hundred other journals, and that's where it's published, I have doubts about the quality of the peer review.

While these new journals face challenges—how to attract good quality submissions, and how to ensure that these submissions are reviewed by trustworthy reviewers—the previous comment shows a strong undercurrent of overflow. New journals' lack of reputation makes them not trusted by scientists, and because of the number of new journals, scientists rarely read what they don't trust, so establishing a reputation is extremely hard. The multiple new journals, and all the papers they contain, add greatly to the overflow.

## Discussion

Two themes emerged from our interview data: (1) the overflow in science is leading to concerns about quality of scientific outputs, (2) scientists often use reputation—of their colleagues or of a journal, for example—as a proxy for trustworthiness. Together, these results reveal a serious issue in the way our interviewees assess the quality of science. Many papers are written by scientists and labs that are simply not known to the established scientists. Similarly, much research is now published in new journals that lack an established track record. In each case reputational judgments of rigour and scientific importance are impossible. This lack of familiarity can lead to an unjustified lack of trust. The growing tension within some areas of science in recent years might be partly motivated by lack of familiarity, and a consequent inability to use professional reputations as a measure of trustworthiness, caused by overflow in the system.

The interviewees clearly stated an aspiration to maintain objectivity when assessing individual papers, while maintaining that they consider the personal reputations of authors to be important. There is a clear contradiction between these positions. Recent initiatives to introduce double-blind refereeing offer an apparent increase in objectivity, but this comes at the cost of a diminished ability for referees to assess the trustworthiness of authors (see, for example, Nature, 2015). As overflow becomes more pronounced, this conflict becomes more problematic. The need for objective treatment of authors increases as more papers are submitted, but at the same time referees necessarily increase their reliance on subjective proxies such as reputation. It is very hard to see how these opposing forces can be reconciled in the current system.

## Have attempts to manage overflow caused problems?

The day-to-day experience of scientists is filled with devices aimed at managing overflow. Most obvious are the impact factors of journals, which are widely agreed to be unrepresentative (Nature, 2005; Garfield, 2006), yet they are widely used as proxies for scientific importance in decisions about hiring and funding. Even more pernicious measures are becoming widespread, such as moves to assess the performance of academic staff by measuring the amount of grant money they bring in (Bishop, 2014; Jump, 2015).

The use of misleading metrics to measure achievement, in the place of careful judgment of cases on their individual merits, clearly causes unfairness in decision-making. It also leads to ‘game-playing’, in which scientific priorities and reporting are aimed exclusively at increasing metric scores. However, this is inevitable when the metrics are adopted ad hoc, without explicitly recognising overflow and assessing how it should best be handled.

## How can overflow in science be managed to increase trustworthiness?

Reducing overflow is hardly a solution. It is widely accepted that funding scientific research leads to many social and economic benefits, and calls to limit science participation are rarely supported by either governments or most scientists (Alberts, 2010; NIH, 2012; Kimble et al., 2015). Successful answers can only lie in managing the quality and assessment of the new science produced. We put forward two general solutions.

Above all, what is badly missing from current discussions is a proper understanding of the nature of the overflow in science. Scientists and policymakers need to be aware of the existence of overflow, of the problems it causes to the body of science, and how it conflicts with solutions to problems such as irreproducibility. We urgently need to understand how great the overflow is, and where it comes from.

The other clear need is for changes in peer review, which either needs to be updated to cope with the demands imposed by overflow or be used less. Others have proposed changes to address, for example, fairness or reproducibility—double-blind peer review, for example. These solutions will likely fail unless they explicitly consider overflow, because scientists will always require reputation-based proxies to guide them through the overflow of information. We therefore suggest two ideas that should be considered by the scientific community.

First, create a system for detailed policing of data quality by non-academic scientists—professionals whose job it is to check experimental design, statistics, analysis, and fraud in images—before referees become involved. This is a version of the triage mechanisms that allow news editors and clinicians to cope with excess demand. It would ensure a basic level of research soundness, and help scientists choose which journals to trust with their submitted papers. It would be also be slow and expensive, but arguably it would be money well spent if it increased the reliability and value of research outputs.

A second, more radical solution to overflow would be to dispense with peer review altogether for a subset of published research. This option is currently unpalatable and unacceptable to the community; peer review is seen as a gold standard that separates acceptable science from hearsay. But as overflow increases, the quality and objectivity of peer review are plainly being eroded (Eisen, 2011), leading to the current state of diminishing trust in science (Steen, 2011; Resnik and Dinse, 2013). As time passes and overflow increases, this erosion can only get worse. Possible alternatives include much wider use of pre-publication archives like arXiv or bioRxiv (Vale, 2015), the replacement of some peer review with vetting by professional editors, shifting towards post-publication commentary fora such as PubPeer, and a shifted emphasis towards depositing primary data and writing fewer, more influential publications.

ILLUSTRATION: CLAUDIA STOCKER, VIVIDBIOLOGY.COM.

Overflow and its consequences are clearly with us to stay. It is hard to anticipate which solutions would best regain the trust of the field. But it must be considered and addressed, and soon, or trust will continue to drain out of the scientific enterprise.
